# Poly(ADP-ribose) Polymerase 1 Mediates Rab5 Inactivation after DNA Damage

**DOI:** 10.3390/ijms23147827

**Published:** 2022-07-15

**Authors:** Masato Mashimo, Akane Morozumi, Akari Nobeyama, Misato Kanzaki, Shigeru Negi, Jiro Kato, Joel Moss, Atsuo Nomura, Takeshi Fujii

**Affiliations:** 1 Laboratory of Pharmacology, Department of Clinical Pharmacy, Faculty of Pharmaceutical Sciences, Doshisha Women’s College of Liberal Arts, Kyotanabe 610-0395, Kyoto, Japan; akane9769@gmail.com (A.M.); lpt02525@gmail.com (A.N.); ykk029@dwc.doshisha.ac.jp (M.K.); a-nomura@dwc.doshisha.ac.jp (A.N.); tfujii@dwc.doshisha.ac.jp (T.F.); 2 Laboratory of Molecular Biophysical Chemistry, Department of Clinical Pharmacy, Faculty of Pharmaceutical Sciences, Doshisha Women’s College of Liberal Arts, Kyotanabe 610-0395, Kyoto, Japan; snegi@dwc.doshisha.ac.jp; 3 Pulmonary Branch, National Heart, Lung, and Blood Institute, National Institutes of Health, Bethesda, MD 20892, USA; katoj@nhlbi.nih.gov (J.K.); mossj@nhlbi.nih.gov (J.M.)

**Keywords:** endocytosis, parthanatos, poly(ADP-ribose) polymerase, Rab5

## Abstract

Parthanatos is programmed cell death mediated by poly(ADP-ribose) polymerase 1 (PARP1) after DNA damage. PARP1 acts by catalyzing the transfer of poly(ADP-ribose) (PAR) polymers to various nuclear proteins. PAR is subsequently cleaved, generating protein-free PAR polymers, which are translocated to the cytoplasm where they associate with cytoplasmic and mitochondrial proteins, altering their functions and leading to cell death. Proteomic studies revealed that several proteins involved in endocytosis bind PAR after PARP1 activation, suggesting endocytosis may be affected by the parthanatos process. Endocytosis is a mechanism for cellular uptake of membrane-impermeant nutrients. Rab5, a small G-protein, is associated with the plasma membrane and early endosomes. Once activated by binding GTP, Rab5 recruits its effectors to early endosomes and regulates their fusion. Here, we report that after DNA damage, PARP1-generated PAR binds to Rab5, suppressing its activity. As a result, Rab5 is dissociated from endosomal vesicles, inhibiting the uptake of membrane-impermeant nutrients. This PARP1-dependent inhibition of nutrient uptake leads to cell starvation and death. It thus appears that this mechanism may represent a novel parthanatos pathway.

## 1. Introduction

Parthanatos is a poly(ADP-ribose) polymerase1 (PARP1)-dependent programmed cell death, which occurs after DNA damage [1,2,3]. PARP1 contains zinc-finger domains that recognize DNA strand breaks and initiate poly(ADP-ribosyl)ation of nuclear proteins, including histones and PARP1 itself [4,5]. When the damage is mild, this poly(ADP-ribosyl)ation of nuclear proteins contributes to DNA repair by recruiting the repair machinery, and is rapidly terminated by the action of nuclear PAR-degrading enzymes such as poly(ADP-ribose) glycohydrolase (PARG) and ADP-ribosyl-acceptor hydrolase 3 (ARH3) [6,7]. On the other hand, when DNA damage is severe, some of the excess poly(ADP-ribose) (PAR) synthesized by overactivated PARP1 is cleaved by PARG, generating protein-free PAR polymer, which is subsequently translocated to the cytoplasm [8,9,10]. In the cytoplasm, PAR binds non-covalently to cytoplasmic and mitochondrial proteins, including apoptosis-inducing factor (AIF), leading to its release from the mitochondrial membrane [3,10,11]. Because AIF possesses a nuclear localization sequence, it is translocated to the nucleus where it interacts with DNase, resulting in large-scale DNA fragmentation and cell death [12]. In addition, PAR also interacts with hexokinase, a key glycolytic enzyme [13,14]. Binding to PAR abolishes hexokinase activity, which results in energy collapse and cell death. Parthanatos is reportedly involved in the cell death seen in heart failure, Parkinson’s disease, and cerebral ischemia-reperfusion injury [15,16,17,18,19], suggesting that PARP1 inhibitors are potential therapeutic agents for those diseases.

Endocytosis, the process by which cells take up membrane-impermeant substances from the extracellular space through vesicular transport, plays important roles in intracellular signaling, nutrient uptake, immune responses and cell motility, as well as in pathological processes such as tumorigenesis [20,21]. Following uptake, some endosomal vesicles are transported to lysosomes for degradation, while the others are returned to the plasma membrane. The destination of these vesicles is determined by the Rab family of small G proteins [22], activation of which is controlled by switching the binding of GDP for GTP. Membrane insertion of Rab requires irreversible modification of the two carboxyl-terminal cysteines with an isoprenyl lipid (geranylgeranyl) moiety [23]. Within the cytoplasm, the GDP dissociation inhibitor (GDI) binds to prenylated Rab in its GDP-bound form to cover the isoprenyl anchor, which keeps Rab in the cytoplasm [24]. GDI-displacement factor dissociates the GDI-Rab complex, allowing Rab’s prenyl anchor to insert into the plasma membrane. Rab is then activated by guanosine nucleotide exchange factor (GEF), which exchanges the bound GDP with GTP, enabling it to fulfill its membrane trafficking function. A specific GTPase-activating protein then mediates Rab inactivation by enhancing the hydrolysis of bound GTP into GDP. The inactive, GDP-bound Rab is then extracted from the membrane by GDI and recycled for another round of function. In its GTP-bound form, Rab5 associates with clathrin-coated pit vesicles, mediating their progression to early endosomes (EEs), and recruits the Rab5 effectors Rabaptin5, Rabex5, early endosomal antigen-1 (EEA1) and Rabenosyn5 [22]. These effectors are involved in the sustained activation of Rab5 and the fusion and expansion of EEs. As EEs mature, Rab5 is replaced by Rab7, which mediates transition of EEs into late endosomes (LEs), which then fuse with lysosomes.

Recent studies have shown that proteins interact with PAR polymers through a PAR-binding motif (PBM), PAR-binding domain (PBD) or WWE domain [25,26]. In addition, proteomic studies have shown that several Rab proteins, including Rab5, associate with PARP1-generated PAR following DNA damage [25]. In the present study, we investigated whether PARP1 activation following DNA damage affects endocytosis. As parthanatos participates in several diseases, the precise molecular mechanism is expected to contribute to the elucidation of the pathology induced by parthanatos.

## 2. Results

### 2.1. PARP1 Mediates Inhibition of OVA Uptake

**Figure 1:**
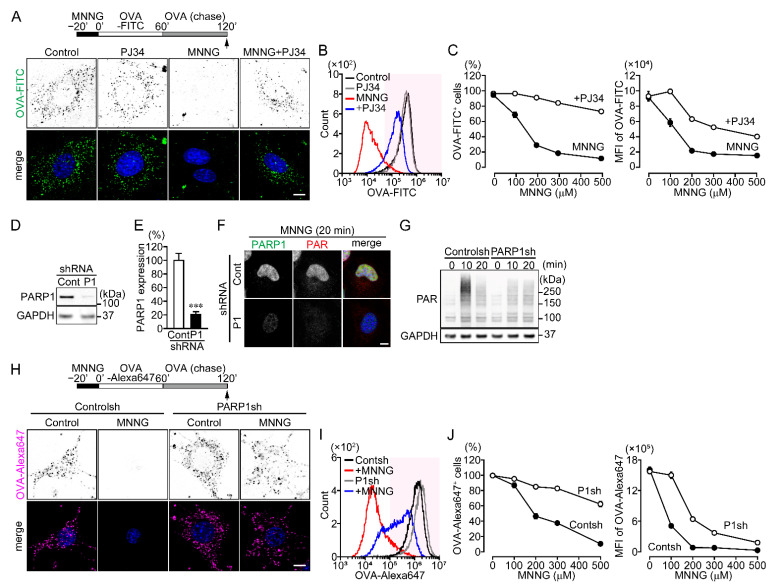
PARP1 activation inhibits OVA uptake following DNA damage. (**A**) OVA-FITC uptake following exposure to MNNG. NIH3T3 cells were pretreated with or without PJ34 (10 μM) for 10 min, then exposed to MNNG (500 μM, 20 min) before incubation with OVA-FITC (50 μg/mL, 1 h) (green) and chased with OVA (100 μg/mL, 1 h). Nuclei were stained with DAPI (blue). Scale bar: 10 μm. (**B**) Histogram of OVA-FITC^+^ cells. After treating cells as in panel (**A**), OVA-FITC^+^ cells (magenta region) were counted using flow cytometry. (**C**) MNNG concentration-dependent inhibition of OVA-FITC uptake (the percentages of OVA-FITC^+^ cells (left) and mean fluorescence intensity (MFI) of OVA-FITC in NIH3T3 cells (right)). Cells were treated as in panel (**A**). Shown are means ± SEM (*n* = 3). *p* < 0.001 at >100 μM. (**D**) PARP1 expression in NIH3T3 expressing PARP1 shRNA. (**E**) Relative PARP1 expression levels. PARP1 protein levels were normalized to GAPDH. Shown are means ± SEM (*n* = 3). *** *p* < 0.001. (**F**) Effect of PARP1 shRNA on PARP1 expression and MNNG-induced PAR synthesis. After exposure to MNNG (500 μM, 20 min), NIH3T3 cells were stained with anti-PARP1 (green) and anti-PAR (red) antibodies. Nuclei were stained with DAPI (blue). Scale bar: 10 μm. (**G**) MNNG-induced PAR synthesis. After exposure to MNNG (500 μM) for the indicated times, NIH3T3 cells were subjected to Western blotting using the indicated antibodies. (**H**) OVA-Alexa647 uptake following exposure to MNNG. NIH3T3 cells stably expressing control or PARP1 shRNA were exposed to MNNG (500 μM, 20 min) before incubation with OVA-Alexa647 (50 μg/mL, 1 h) (magenta) and then chased with OVA (100 μg/mL, 1 h). Nuclei were stained with DAPI (blue). Scale bar: 10 μm. (**I**) Histogram of OVA-Alexa647^+^ cells. After the same procedure described for panel (**H**), OVA-Alexa647^+^ cells (magenta region) were counted using flow cytometry. (**J**) MNNG concentration-dependent inhibition of OVA-Alexa647 uptake (the percentages of OVA-Alex647^+^ cells (left) and MFI of OVA-Alexa647 in NIH3T3 cells (right)). The procedure was the same as in panel (**H**). Shown are means ± SEM (*n* = 3). *p* < 0.05 at >100 μM. Data information: Panels (**C**,**J**): two-way ANOVA with post hoc Tukey’s test; (**E**): Student’s *t*-test.

To investigate whether poly(ADP-ribosyl)ation catalyzed by PARP1 regulates endocytic events, the DNA alkylating agent N-methyl-N’-nitro-N-nitrosoguanidine (MNNG) (500 μM, 20 min) was added to NIH3T3 and HeLa cells to induce PARP1 activation before addition of fluorescently labelled ovalbumin (OVA-FITC and OVA-Alexa647). MNNG dose-dependently inhibited the percentage of NIH3T3 cells that took up OVA-FITC and the amount of OVA-FITC taken up cells, which was reversed by pretreatment with the PARP inhibitors PJ34, ABT-888 or XAV-989 (Figure 1A–C and Figure S1A). To assess the involvement of PARP1, we prepared NIH3T3 and HeLa cells stably expressing control or PARP1 shRNA. In both cell types, PARP1 shRNA reduced PARP1 expression by approximately 80% compared to control and suppressed MNNG-induced nuclear PAR synthesis (Figure 1D–G and Figure S1B–E). OVA-Alexa647 uptake following exposure to MNNG was partially restored by expression of PARP1 shRNA (Figure 1H–J and Figure S1F,G). Thus, PARP1 activation is required for inhibition of OVA uptake after DNA damage.

**Figure 2:**
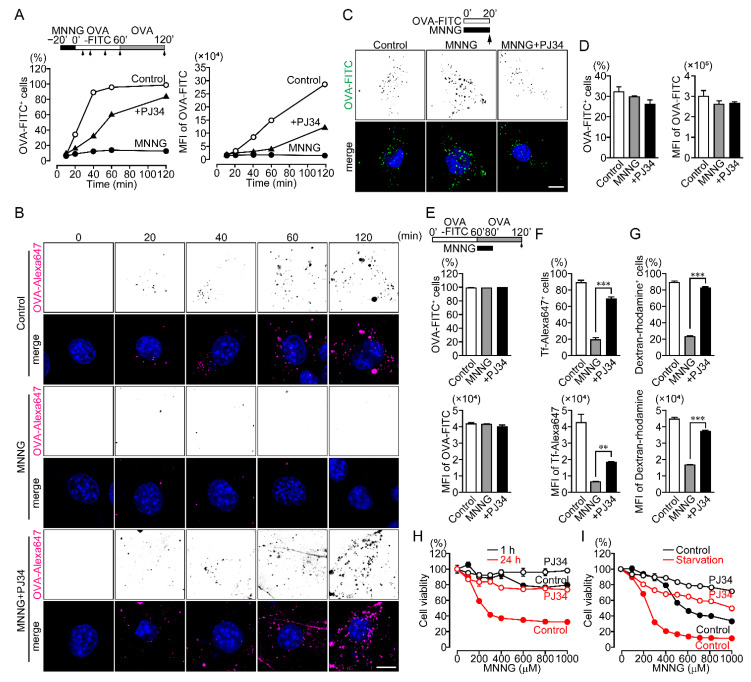
PARP1-dependent inhibition of endocytosis results in cell death. (**A**) Time course of OVA-FITC uptake (the percentages of OVA-FITC^+^ cells (left) and MFI of OVA-FITC in NIH3T3 cells (right)). NIH3T3 cells were pretreated with or without PJ34 (10 μM) for 10 min, then exposed to MNNG (500 μM, 20 min), after which they were incubated with OVA-FITC (50 μg/mL) then chased with OVA (100 μg/mL) for the indicated times. Shown are means ± SEM (*n* = 3). *p* < 0.05 at >20 min (left) and >60 min (right). (**B**) Subcellular localization of OVA-Alexa647. NIH3T3 cells were pretreated with or without PJ34 (10 μM) for 10 min and then exposed to MNNG (500 μM, 20 min) before incubation with OVA-Alexa647 (50 μg/mL) and chased with OVA (100 μg/mL) for the indicated times. Scale bar: 10 μm. (**C**) OVA-FITC uptake following simultaneous exposure to MNNG and OVA-FITC. NIH3T3 cells were pretreated with or without PJ34 (10 μM) for 10 min, then exposed to MNNG (500 μM) and OVA-FITC (50 μg/mL, green) for 20 min. Nuclei were stained with DAPI (blue). Scale bar: 10 μm. (**D**) Effect of simultaneous addition of MNNG and OVA-FITC on OVA-FITC uptake. With the same procedure as in panel (**C**), OVA-FITC^+^ cells (left) and MFI of OVA-FITC (right) were counted using flow cytometry. Shown are means ± SEM (*n* = 3). (**E**) Effect of endosomal recycling on OVA-FITC uptake (the percentages of OVA-FITC^+^ cells (upper) and MFI of OVA-FITC in NIH3T3 cells (lower)). After incubation with OVA-FITC (50 μg/mL, 1 h), NIH3T3 cells were exposed to MNNG (500 μM, 20 min) with OVA (100 μg/mL, 1 h). Shown are means ± SEM (*n* = 3). (**F**) Tf-Alexa647 uptake (the percentages of Tf-Alexa647^+^ cells (upper) and MFI of Tf-Alexa647 in NIH3T3 cells (lower)). NIH3T3 cells were exposed to MNNG (500 μM, 20 min) before incubation with Tf-Alexa647 (15 μg/mL, 1 h). Shown are means ± SEM (*n* = 3).** *p* < 0.01, *** *p* < 0.001 (**G**) Dextran-rhodamine uptake (the percentages of Dextran-rhodamine^+^ cells (upper) and MFI of Dextran-rhodamine in NIH3T3 cells (lower)). NIH3T3 cells were exposed to MNNG (500 μM, 20 min) before incubation with dextran-rhodamine (50 μg/mL, 1 h). Shown are means ± SEM (*n* = 3). *** *p* < 0.001 (**H**) Short- and long-term cytotoxic effects of MNNG. NIH3T3 cells were pretreated for 10 min with or without PJ34 (10 μM) before cell viability was measured after 1-h (black) or 24-h (red) exposure to MNNG at the indicated concentrations. Shown are means ± SEM (*n* = 3). *p* < 0.001 between 1-h and 24-h at concentrations > 100 μM in control group. (**I**) MNNG-induced cytotoxicity during starvation. NIH3T3 cells were pretreated for 10 min with or without PJ34 (10 μM), then exposed to MNNG (20 min) at the indicated concentrations before incubation for 24 h in serum-containing (black) or serum-free (red) medium. Shown are means ± SEM (*n* = 3). *p* < 0.001 between control and starvation at concentrations >100 μM in the control group. Data information: Panels (**A**,**H**,**I**): two-way ANOVA with post hoc Tukey’s test; (**D**–**G**): one-way ANOVA with post hoc Tukey’s test.

The inhibition of OVA uptake developed at least 20 min after exposure to MNNG in NIH3T3 cells (Figure 2A,B). Simultaneous addition of MNNG with OVA-FITC did not affect OVA uptake (Figure 2C,D), suggesting PARP1-mediated inhibition of OVA uptake requires minutes to develop. Once a substance is taken up by endocytosis, a portion of it is returned to the extracellular environment via endosomal recycling. To determine whether PARP1 activation accelerates endosomal recycling, OVA-FITC was added to NIH3T3 cells for 1 h before addition of MNNG. Exposure to MNNG after OVA-FITC uptake did not affect the proportion of cells that contained OVA-FITC (Figure 2E), indicating that PARP1 inhibits OVA uptake rather than promoting the recycling pathway. Uptake of OVA as well as transferrin (Tf) is mainly via receptor-mediated endocytosis and macropinocytosis, while uptake of smaller molecules (e.g., dextran) is accomplished by macropinocytosis [27]. PARP1 also inhibited uptake of both Tf-Alexa647 and dextran-rhodamine (Figure 2F,G), indicating that PARP1 activation following exposure to MNNG inhibits receptor-mediated endocytosis and macropinocytosis.

Because MNNG-induced PARP1 activation triggers parthanatos, we assessed the effect of PARP1-mediated inhibition of OVA uptake on cell viability. NIH3T3 cells exposed to MNNG for 24 h exhibited diminished viability, and this effect was suppressed by pretreatment with the PARP1 inhibitor PJ34 (Figure 2H). In contrast, exposure to MNNG for 1 h was rarely cytotoxic (Figure 2H). This suggests PARP1-mediated inhibition of OVA uptake occurs prior to cell death, as parthanatos only occurs after long-term exposure to MNNG.

Endocytosis contributes to cell survival by supplying cells with nutrients and macromolecules that cannot permeate the cell membranes and by controlling receptor signaling to adapt cellular stress [20,21]. When NIH3T3 cells were starved in serum-free Dulbecco’s modified Eagle’s medium (DMEM), exposure to MNNG exacerbated the decline in cell viability as compared to cells in DMEM with 10% fetal bovine serum (FBS) (Figure 2I). Thus, PARP1-mediated inhibition of endocytosis results in cell death, which may be a novel pathway in PARP1-dependent parthanatos.

**Figure 3:**
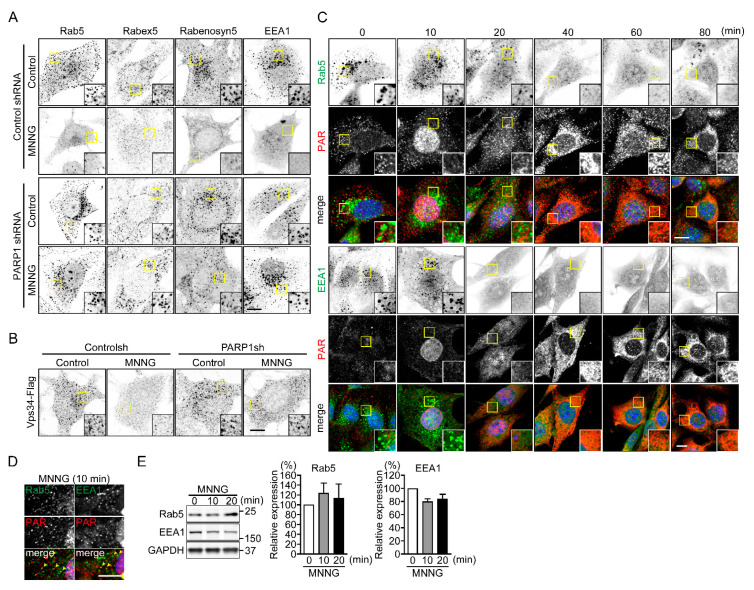
PARP1 dissociates Rab5 and its effector proteins from EE. (**A**) Subcellular localization of Rab5 and its effector proteins following exposure to MNNG. After exposure to MNNG (500 μM, 20 min), NIH3T3 cells were stained with the indicated antibodies. Scale bar: 10 μm. (**B**) Subcellular localization of Vps34-Flag following exposure to MNNG. After exposure to MNNG (500 μM, 20 min), NIH3T3 cells were stained with anti-Flag antibodies. Scale bar: 10 μm. (**C**) Time-dependent changes in subcellular localization of PAR, Rab5 and EEA1 following exposure to MNNG. After exposure to MNNG (500 μM), NIH3T3 cells were stained with anti-Rab5 (green in left panels) or anti-EEA (green in right panels) and anti-PAR (red) antibodies. Nuclei were stained with DAPI. Scale bar: 10 μm. (**D**) Cytoplasmic PAR localization. After exposure to MNNG (500 μM, 10 min), NIH3T3 cells were stained with anti-Rab5 (green, left panel) or anti-EEA1 (green, right panel) and anti-PAR (red) antibodies. Arrowheads indicate colocalization of PAR and Rab5 or EEA1 within the cytoplasm. Nuclei were stained with DAPI. Scale bar: 10 μm. (**E**) Expression of early endosomal proteins (Rab5 (left) and EEA1 (right)). After exposure to MNNG (500 μM) for indicated times, NIH3T3 cells were subjected to Western blotting using indicated antibodies. Protein levels were normalized to GAPDH. Shown are means ± SEM (*n* = 3). Data information: Panel (**E**): one-way ANOVA with post hoc Tukey’s test.

To identify the molecular mechanism underlying PARP1-mediated inhibition of OVA uptake, we observed the subcellular localization of endocytosis-related proteins (Figure S2A). Exposure to MNNG altered the distribution of the early endosomal proteins (Rab5, Rabex5, Rabenosyn5 and EEA1), but not late-endosomal (Rab7) or lysosomal proteins (Lamp1 and 2 and Cathepsin S), from a punctate pattern to uniform distribution in the cytoplasm (Figure 3A and Figure S2B). Stable expression of PARP1 shRNA, or pretreatment with PJ34, suppressed the MNNG-induced changes in the localization of early endosomal proteins (Figure 3A and Figure S2B). The distribution of Flag-tagged Vps34, a class III phosphatidylinositol (PI)-3 kinase, was also altered upon PARP1 activation (Figure 3B and Figure S2C). Time-lapse imaging of red fluorescent protein (RFP)-EEA1 and blue fluorescent protein (BFP)-Rab5 after DNA damage induced by MNNG (Figure S3A,B) or laser micro-irradiation of the nuclei (Figure S3C,D) revealed their similar redistribution, which was suppressed by pretreatment with PJ34.

The changes in the subcellular localization of Rab5 and EEA1 appeared 20 min after exposure to MNNG, which was consistent with the time required for PAR synthesis in the nucleus and its translocation to the cytoplasm (Figure 3C). Moreover, after 10-min exposure to MNNG, some punctate PAR signals in the cytoplasm were colocalized with Rab5 and EEA1 (Figure 3D). Western blot analysis revealed that exposure to MNNG did not induce degradation of these endosomal proteins (Figure 3E). Instead, it enhanced their dissociation from EEs.

### 2.2. PAR Binds to the PBM of Rab5 in Cells Exposed to MNNG

**Figure 4:**
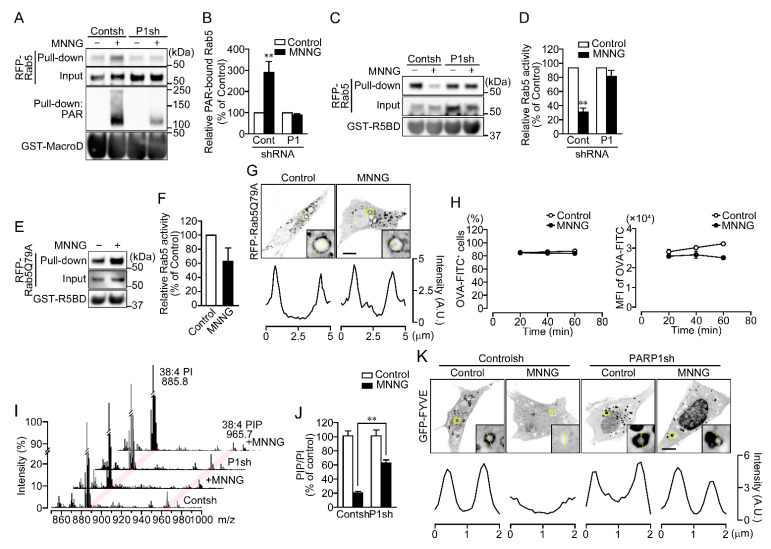
Rab5 is inactivated through PAR binding. (**A**) PAR-binding. After exposure to MNNG (100 μM, 10 min), HeLa cells expressing RFP-Rab5 were subjected first to pull-down assays using GST-Af1521 macrodomain and then to Western blotting using the indicated antibodies. (**B**) Relative levels of PAR-bound Rab5. Ratios of PAR-bound Rab5 (pull-down) to Rab5 (input) were normalized to control. Shown are means ± SEM (*n* = 4). ** *p* < 0.01 vs. control. (**C**) Rab5 activity. After exposure to MNNG (100 μM, 20 min), HeLa cells expressing RFP-Rab5 were subjected first to pull-down assay using R5BD-GST and then to Western blotting using the indicated antibodies. (**D**) Relative Rab5 activity. Ratios of GTP-bound Rab5 (pull-down) to Rab5 (input) were normalized to control. Shown are means ± SEM (*n* = 3). ** *p* < 0.01 vs. control. (**E**) Rab5 activity of the Rab5Q79A mutant. After exposure to MNNG (100 μM, 20 min), HeLa cells expressing Rab5Q79A were subjected first to pull-down assays using GST-R5BD and then to Western blotting using anti-mCherry and anti-GST antibodies. (**F**) Relative Rab5 activity of the Rab5Q79A mutant. Ratios of GTP-bound Rab5 (pull-down) to Rab5 (input) were normalized to control. Shown are means ± SEM (*n* = 3). (**G**) Subcellular localization of Rab5Q79A. HeLa cells expressing Rab5Q79A were exposed to MNNG (100 μM, 20 min). Graphs show the fluorescence intensity of RFP-Rab5Q79A on the dashed yellow lines in the magnified images. (**H**) Effect of the Rab5Q79A mutant on OVA-FITC uptake following exposure to MNNG. HeLa cells expressing RFP-Rab5Q79A were exposed to MNNG (100 μM, 20 min) before incubation with OVA-FITC (50 μg/mL, 1 h). OVA-FITC^+^ cells (left) and MFI of OVA-FITC in HeLa cells (right) were counted using flow cytometry. Shown are means ± SEM (*n* = 3). *p* < 0.05 at 40 and 60 min in MEF of OVA-FITC (**I**) PI and PIP levels in membrane fractions. After exposure to MNNG (500 μM, 20 min), membrane fractions were extracted from NIH3T3 cells. Two peaks (*m*/*z* 885.8 and 965.7) presumed to be PI (38:4) and PIP (38:4), respectively, are indicated by magenta lines. PIP levels were normalized by the peak intensity of PI. (**J**) Relative PIP/PI levels. Ratios of PI to PIP were normalized to control. Cells were pretreated for 10 min with or without PJ34 (10 μM). Shown are means ± SEM (*n* = 3). ** *p* < 0.01 (**K**) Subcellular localization of GFP-FYVE. NIH3T3 cells were pretreated for 10 min with or without PJ34 (10 μM), then exposed to MNNG (500 μM, 20 min). Graphs show the fluorescence intensity of GFP-FYVE on the dashed yellow lines in the magnified images. Scale bar: 10 μm. Data information: Panels (**B**,**D**,**H**,**J**): two-way ANOVA with post hoc Tukey’s test; (**F**): Student’s *t*-test.

Because only proteins localized in EEs were dissociated upon PARP1 activation, we investigated whether Rab5, a master regulator of early endosomal fusion, is inactivated through PAR binding. To detect PAR-Rab5 binding, pull-down assays using GST-Af1521 macrodomain were performed to isolate PAR-bound proteins (Figure S4A) [28]. Assays using GST-Af1521 macrodomain, but not GST, revealed PAR binding to RFP-Rab5 in HeLa cells exposed to MNNG for 10 min (Figure 4A,B and Figure S4B). The interaction was blocked, when PAR synthesis was suppressed by expression of PARP1 shRNA or pretreatment with PJ34 (Figure 4A,B and Figure S4B–D). To test whether PAR binding blocks Rab5 activity, the levels of GTP-bound Rab5 were measured in pull-down assays using GST-Rab5-binding domain (R5BD) from Rabaptin5 (Figure S4E) [29]. The assays revealed that levels of active RFP-Rab5 were diminished in cells exposed to MNNG for 10 min (Figure 4C,D and Figure S4F–H). By contrast, RFP-Rab5Q79A, a dominant-active Rab5 mutant, remained in the GTP-bound form in those cells (Figure 4E,F). Exposure to MNNG also did not affect the subcellular localization of RFP-Rab5Q79A (Figure 4G). Moreover, expression of RFP-Rab5Q79A slightly reduced the amount of OVA-FITC incorporated into cells following exposure to MNNG, but did not alter the percentage of cells that took up OVA-FITC (Figure 4H). These results indicate that PARP1-generated PAR binds Rab5 in the cytoplasm, thereby suppressing GDP-to-GTP exchange and resulting in loss of Rab5 activity.

GTP-bound Rab5 recruits Vps34 to EEs where it catalyzes the phosphorylation of PI to phosphatidylinositol-3-phosphate (PI3P) [30]. PI3P in endosomal vesicles recruits EEA1 and rabenosyn5, which contains an FYVE domain [31]. Because PARP1 activation also leads to dissociation of Vps34 from EEs along with Rab5 (Figure 3B), we tested whether phosphatidylinositol phosphate (PIP) levels in membrane fractions were reduced by MNNG. Electrospray ionization mass spectrometry revealed that exposing cells to MNNG for 20 min led to PARP1-dependent decreases in the PIP-to-PI ratio (Figure 4I,J and Figure S4I). Consistent with that finding, MNNG induced dissociation of green fluorescent protein (GFP)-FYVE, a PI3P marker, from EEs (Figure 4K and Figure S4J), suggesting that PARP1 activation reduces PI3P levels in EEs.

Inhibition of Rab5 insertion into the membrane of vesicles by GDI [24] appears to be regulated by several kinases, including p38 mitogen-activated protein kinase (MAPK) [32]. Inhibitors of p38 MAPK (SB 203580), mitogen-activated protein kinase (MEK)(U126), AMP-activated protein kinase (AMPK)(dorsomorphin) and mammalian target of rapamycin (mTOR)(Rapamycin) did not suppress MNNG-induced inhibition of OVA uptake (Figure S5A,B). Moreover, MNNG did not change the subcellular localization of GDI1 or 2 (Figure S5C). Thus, PARP1-dependent inhibition of endocytosis does not result from inactivation of Rab5 by GDI, but from inhibition of Rab5 activation.

**Figure 5:**
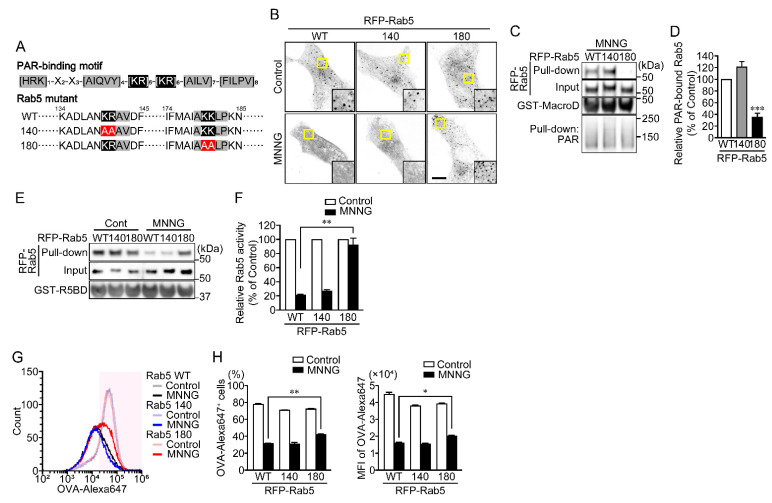
PAR binds the PAR-binding motif near amino acid residue 180 inactivates Rab5. (**A**) Putative PAR-binding motif in Rab5. Arginine and lysine residues within the PAR-binding motif were replaced with alanine. (**B**) Subcellular localization of RFP-Rab5 and its mutants. HeLa cells transiently expressing RFP-Rab5 WT or its mutants were exposed to MNNG (100 μM, 20 min). Scale bar: 10 μm. (**C**) PAR-binding assay. After exposure to MNNG (100 μM, 20 min), HeLa cells expressing RFP-Rab5 WT or its mutants were subjected first to pull-down assays using GST-Af1521 macrodomain and then to Western blotting using the indicated antibodies. (**D**) Relative PAR-bound Rab5 levels. Ratios of PAR-bound Rab5 (pull-down) to Rab5 (input) were normalized to control. Shown are means ± SEM (*n* = 3). *** *p* < 0.001 vs. WT. (**E**) Rab5 activity assay. After exposure to MNNG (100 μM, 20 min), HeLa cells expressing RFP-Rab5 WT or its mutants were subjected to first pull-down assays using R5BD-GST and then to Western blotting using the indicated antibodies. (**F**) Relative Rab5 activity. Ratios of GTP-bound Rab5 (pull-down) to Rab5 (input) were normalized to control. Shown are means ± SEM (*n* = 3). ** *p* < 0.01. (**G**) Histogram of OVA-Alexa647^+^ cells. HeLa cells expressing RFP-Rab5 WT or its mutants were exposed to MNNG (100 μM, 20 min) before incubation with OVA-FITC (50 μg/mL, 1 h) (green) and then chased with OVA (100 μg/mL, 1 h). OVA-Alexa647^+^ cells (within magenta region) were counted using flow cytometry. (**H**) Effect of Rab5 mutations on OVA-Alexa647 uptake (the percentages of OVA-Alexa647^+^ cells (left) and MFI of OVA-Alexa647 in HeLa cells (right)) following exposure to MNNG. Shown are means ± SEM (*n* = 3). * *p* < 0.05, ** *p* < 0.01. Data information: Panels (**D**): one-way ANOVA with post hoc Tukey’s test; (**F**,**H**): two-way ANOVA with post hoc Tukey’s test.

A putative PAR-binding motif ([HKR]_1_-X_2_-X_3_-[AIQVY]_4_-[KR]_5_-[KR]_6_-[AILV]_7_-[FILPV]_8_) has been identified through proteomic analysis [25]. Notably, the positively charged amino acids of the 5th and 6th arginine and/or lysine residues ([KR]_5_-[KR]_6_) are important for binding to the negatively charged phosphate group of PAR. Rab5 appears to have two putative PAR-binding motifs located at around amino acid residues 140 and 180, respectively (Figure 5A). To identify the PAR-binding site on Rab5, RFP-Rab5 K140AK141A (Rab5 140 mutant) and K180AR181A (Rab5 180 mutant) mutants were prepared. Like RFP-Rab5 wild-type (WT), both the Rab5 140 and 180 mutants showed a punctate distribution in the cytoplasm under unstimulated conditions (Figure 5B). However, MNNG induced dissociation of Rab5 WT and the Rab5 140 mutant from EEs, whereas the Rab5 180 mutant remained in EEs (Figure 5B). In contrast to RFP-Rab5 WT and the Rab5 140 mutant, the RFP-Rab5 180 mutant did not bind PAR, and levels of the GTP-bound form were unaffected by MNNG (Figure 5C–F). In addition, expression of the RFP-Rab5 180 mutant restored OVA-Alexa647 uptake in cells exposed to MNNG (Figure 5G,H). These results indicate that PAR, generated by PARP1 in response to DNA damage, and then translocated to the cytoplasm, binds the PAR-binding motif near amino acid residue 180 of Rab5, which results in loss of its activity.

### 2.3. Rab5 and Its Effectors Are Dissociated from EEs in Neuronal Cells after PARP1 Activation

**Figure 6:**
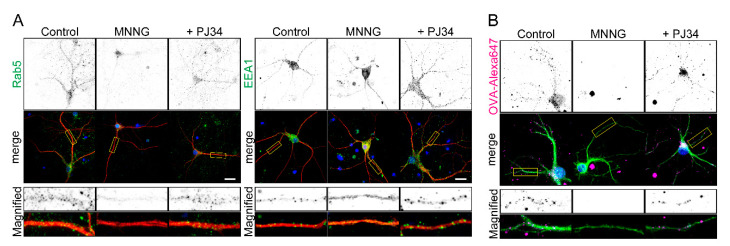
PARP1 inhibits endocytosis by dissociating Rab5 and EEA1 in hippocampal primary neurons. (**A**) Subcellular localization of Rab5 an EEA1 within neurites. Primary hippocampal neurons were pretreated for 10 min with or without PJ34 (10 μM), then exposed to MNNG (500 μM, 20 min) before staining with anti-MAP2 antibodies (red) and anti-Rab5 (green, left) or anti-EEA1 (green, right). (**B**) OVA-Alexa uptake by primary hippocampal neurons. Neurons were pretreated for 10 min with or without PJ34 (10 μM), then exposed to MNNG (500 μM, 20 min) before addition to OVA-Alexa647 (50 μg/mL, 1 h). Neurites and nuclei were marked with MAP2 (green) and DAPI (blue), respectively. Scale bar: 10 μm.

Parthanatos is frequently the cause of neuronal cell death [1,8]. We therefore tested whether PARP1-dependent inhibition of OVA uptake occurs in primary cultures of hippocampal neurons. Exposure to MNNG resulted in dissociation of Rab5 and EEA1 from EEs within the neurites and soma of neurons (Figure 6A) and inhibition of OVA uptake (Figure 6B). These effects were blocked by pretreatment with PJ34. Thus, under conditions where parthanatos occurs in neurons, PARP1 inhibits nutrient uptake by suppressing EE maturation.

## 3. Discussion

In this study, we found that Rab5 and its effectors dissociate from EEs upon PARP1 activation. Thus, PARP1-dependent suppression of OVA, Tf and dextran uptake after DNA damage apparently reflects the suppression of EE maturation and fusion that results from dissociation of Rab5 and its effectors. This finding is consistent with the fact that, in vivo, in adult mouse liver Rab5 knockdown using siRNA markedly reduces numbers of EEs, late endosomes and lysosomes and is associated with suppression of endocytosis of low-density lipoprotein [33]. After insertion into endosomal membranes, GTP-bound Rab5 binds to Rabaptin5, which recruits Rabex5, a Rab5 GEF, to maintain the GTP-bound state of Rab5 [34,35]. PARP1 activation reduces the GTP-bound state in cells expressing RFP-Rab5 WT but not a constitutively active form of Rab5. We therefore speculate that in the event of DNA damage, PAR produced by PARP1 binds to the PAR-binding motif in its C-terminal region of Rab5, preventing Rab5 from maintaining its GTP-bound state. Rab5 consists of six central β-sheets (β1-6) surrounded by five α-helices (α1-α5) [36,37]. The PAR-binding motif of Rab5 is located within α5-helix, on the surface of the protein, opposite the β-sheet required for the binding Rabaptin5 [34]. The binding of PAR to Rab5 induces a conformational change in the protein that inhibits its binding to Rabaptin5. In fact, because the Rab5-binding domain used to detect GTP-bound Rab5 in this study is derived from Rabaptin5, the results of the GST pull-down assays with R5BD are consistent with the dissociation of the Rab5-Rabaptin5 interaction upon PAR binding. Alternatively, the PAR-binding motif of Rab5 is in close proximity to the two prenylated cysteine residues in the C-terminal region [23], so that PAR binding may also inhibit their modification. As a result, PAR binding may prevent Rab5 from translocating to the EE membrane and inhibit its GEF-mediated GTP exchange.

Vps34 binds to GTP-bound Rab5 and catalyzes the phosphorylation of PI to PI3P in EEs [30]. EEA1 binds to GTP-bound Rab5 and to PI3P in the EE membrane via the FYVE domain, thereby associating EEs with each other [31]. In addition, GTP-bound Rab5 fuses EEs together by accumulating Rabenosyn-5-Vps45 complexes, which modulate the accumulation of vesicle fusion-regulating SNAREs [38]. The dissociation of activated Rab5 from EEs resulted in the dissociation of Vps34 and a decrease in the PI3P content of EEs. This suggests that dissociation of EEA1 and Rabenosyn5 from vesicles follows from the dissociation of Rab5 from EEs.

GDI binds to GDP-bound Rab5 and prevents it from localizing at the membrane. The activity of GDI is regulated by p38 MAPK [32]. The fact that p38 inhibition did not suppress the inhibition of OVA uptake following PARP1 activation, and that GDI localization was unchanged in cells exposed to MNNG, indicates that GDI does not participate in the inactivation of Rab5 or its dissociation from EEs following PARP1 activation.

Endocytosis contributes to cell survival by mediating the uptake of proteins and other membrane-impermeant nutrients [21]. In addition, it controls receptor activation and signaling [39,40]. Endocytosis reduces the number of receptors available for extracellular ligands, which attenuates signaling triggered by the plasma membrane. On the other hand, many types of receptors require endocytosis to interact with downstream effectors for sustained activation of signaling. In this study, we found that PARP1-dependent inhibition of Rab5 activity promotes cell death under starvation conditions. This may result from inhibition of nutrient uptake and receptor signaling involved in cell survival and proliferation, which may be a novel parthanatos pathway. Parthanatos has been implicated in Parkinson’s disease, Alzheimer’s disease, and neuronal death during cerebral ischemia-reperfusion [15,16,17,18,19]. Although the role of PARP1-dependent inhibition of nutrient uptake remains unclear, our findings suggest its potential involvement in the induction of neuronal cell death during these pathological conditions.

## 4. Materials and Methods

### 4.1. Materials

Rabbit monoclonal anti-GAPDH antibody (14C10), rabbit monoclonal anti-mCherry antibody (E5D8F), rabbit monoclonal anti-Rab5 antibody (C8B1), and rabbit polyclonal anti-PARP1 antibody were purchased from Cell Signaling Technology (Danvers, MA, USA). Mouse monoclonal anti-PAR antibody (10H) was from Enzo Life Sciences (Farmingdale, NY, USA). Mouse monoclonal anti-GST antibody, OVA-FITC, OVA-Alexa647, OVA, Tf-Alexa647, dextran-rhodamine B 7000 MW, Lipofectamine 3000, and Alexa Fluor-conjugated secondary antibodies were from Thermo Fisher Scientific (Waltham, MA, USA). HRP-conjugated secondary antibodies were from Promega (Madison, MI, USA). Rabbit polyclonal anti-Rabenosyn5 antibody, rabbit polyclonal anti-GDI1 antibody, and rabbit polyclonal anti-GDI2 antibody were from Proteintech (Rosemont, IL, USA). Rabbit polyclonal anti-EEA1 antibody (H-300), anti-Clathrin heavy chain antibody (A-8), mouse monoclonal anti-Rabex5 antibody (C-4), mouse monoclonal anti-LAMP1 antibody (H4A3), mouse monoclonal anti-LAMP2 antibody (H4B4), mouse monoclonal anti-Cathepsin S antibody (E-3), PJ34, and ADP-HPD were from Santa Cruz Biotechnology (Dallas, TX, USA). SB203580 was from AdipoGen Life Sciences (San Diego, CA, USA). U0126 was from Cayman Chemical Company (Ann Arbor, MI, USA). Rapamycin and dorsomorphin were from Fujifilm (Tokyo, Japan). Mouse monoclonal anti-Flag antibody (OTI4C5) was from Origene (Rockville, MD, USA).

### 4.2. Plasmid Vectors

pET-GST-R5BD-hRABEP1 was purchased from Vector Builder. Rab5a-pmCherryC1 was a gift from Christien Merrifield (Addgene plasmid #27679; http://n2t.net/addgene:27679, accessed on 31 May 2019; RRID:Addgene_27679) [41]. pcDNA4-Vps34-Flag was from Qing Zhong (Addgene plasmid #24398; http://n2t.net/addgene:24398, accessed on 12 November 2018; RRID:Addgene_24398) [42]. mCherry-Rab5CA(Q79L) was from Sergio Grinstein (Addgene plasmid #35138; http://n2t.net/addgene:35138, accessed on 26 April 2018; RRID:Addgene_35138) [43]. TagRFP-T-EEA1 was from Silvia Corvera (Addgene plasmid #42635; http://n2t.net/addgene:42635, accessed on 9 November 2017; RRID:Addgene_42635) [44]. pTag-BFP-C-h-Rab5a-c-Myc was from James Johnson (Addgene plasmid #79801; http://n2t.net/addgene:79801, accessed on 9 November 2017; RRID:Addgene_79801) [45]. RFP-Rab5 140 and 180 mutants were generated with primers (5′-GCAGCAGCTGTTGACTTCCAGG-3′ and 5′-ATTTGCTAAGTCAGCTTTGTTTCCTGAC-3′) and (5′-GCAGCGCTGCCAAAGAATGAAC-3′ and 5′-AGCTATTGCCATAAATATTTCATTTACATTCATTG-3′), respectively, using a KOD-plus mutagenesis kit (Toyobo, Osaka, Japan).

### 4.3. Cell Culture

NIH3T3 and HeLa cells were incubated in DMEM containing 10% FBS, 100 units of penicillin, and 100 μg/mL streptomycin at 37 °C in a humidified atmosphere with 5% CO_2_. shRNA plasmids targeting human or mouse PARP1 or scrambled shRNA (Origene) were introduced into NIH3T3 and HeLa cells using Lipofectamine 3000 transfection reagent according to manufacturer’s instructions. Cells stably expressing shRNA plasmids were selected in medium containing 1 μg/mL puromycin.

Primary hippocampal neurons were isolated using neuron dissociation solution (Sumiron, Osaka, Japan) and cultured in neurobasal medium with B-27 supplement on poly-*d*-lysine-coated dishes. Half the volume of the medium was replaced every 5 days. The protocols used in this study were approved by the Ethical Committee of Doshisha Women’s College of Liberal Arts (Nos. Y15-027, Y16-30, Y17-031).

### 4.4. OVA, Tf and Dextran Uptake

For flow cytometric measurement of the uptake of OVA-FITC, OVA-Alexa647, Tf-Alexa647 or dextran-rhodamine, NIH3T3 or HeLa cells (1 × 10^5^ cells) were seeded onto 24-well plates and incubated for 3 h in serum-free DMEM with non-essential amino acids cell culture supplement (Nacalai Tesque, Kyoto, Japan). PARP inhibitors were added for 10 min before 20-min exposure to MNNG. After washing with PBS, the cells were incubated with OVA-FITC (50 μg/mL), OVA-Alexa 647 (50 μg/mL), Tf-Alexa 647 (15 μg/mL), or dextran-rhodamine B 7000 MW (1.5 μg/mL) for the indicated times. Cells were trypsinized and suspended in PBS. Fluorescence was measured using a Cytoflex flow cytometer. The data were analyzed and processed using Cytexpert (Beckman Coulter, Brea, MA, USA).

### 4.5. Cell Viability Assays

NIH3T3 and HeLa cells (1 × 10^4^ cells) seeded onto 96-well plates were incubated for 30 min with a PARP inhibitor and then exposed to MNNG. Cell numbers were then counted using cell counting reagent SF (Nacalai Tesque) according to the manufacturer’s instructions by measuring the absorbance at 450 nm (SpectraMax M5 Microplate Reader, Molecular Devices, San Jose, CA, USA).

### 4.6. Immunocytochemistry

NIH3T3 and HeLa cells (1 × 10^5^ cells) and primary hippocampal neurons were seeded onto 8-well chamber plates, fixed with 4% paraformaldehyde (PFA; 20 min, 4 °C), permeabilized, and blocked with Blocking One (Nacalai Tesque) containing 0.5% Triton X-100 (30 min, room temperature). After incubation (overnight, 4 °C) with primary antibodies, the cells were treated (1 h, room temperature) with Alexa-488-conjugated goat anti-rabbit IgG or Alexa-564-conjugated goat anti-mouse IgG (1:500), washed three times with PBS, and incubated (5 min, room temperature) with 300 nM DAPI (Thermo Fisher Scientific) to stain the nuclei. The cells were then imaged using a confocal microscope (Zeiss LSM 700 Meta; Carl Zeiss, Jena, Germany) equipped with an oil-immersion objective (60×, numerical aperture = 1.4). Fluorescence data were processed and analyzed using FijiJ.

### 4.7. Live-Cell Imaging after DNA Damage

NIH3T3 cells (1 × 10^5^ cells) seeded onto glass-bottomed dished were transfected using Lipofectamine 3000 according to the manufacturer’s instructions. After incubation for 1 day, fluorescence was observed with a confocal microscope (Zeiss LSM 700 Meta) equipped with an oil-immersion objective (63×, numerical aperture = 1.4). Hoechst 33258 (10 μg/mL; Dojindo, Kumamoto, Japan) was added to enhance DNA damage. Micro-irradiation of whole nuclei was carried out with a 405 nm diode laser set to 100% transmission [46]. The microscope was equipped with a heated environmental chamber set to 37 °C. Images were taken every 30 s for 40 min.

### 4.8. Western Blotting

Cells (3 × 10^5^ cells) seeded onto 6-well plates were incubated (1 day, 37 °C) in DMEM with 10% FBS. Cell lysates were prepared with 2% SDS in 20 mM Tris-HCl (pH 7.4) containing complete protease inhibitor cocktail (Roche, Basel, Switzerland). After adjustment of the protein concentration using a BCA kit (Thermo Fisher Scientific), cell lysates were subjected to Bis-Tris SDS-PAGE (Thermo Fisher Scientific) and then transferred to nitrocellulose membranes (Thermo Fisher Scientific). The membranes were blocked with Blocking One for 30 min at room temperature and then incubated with primary antibodies. After incubation with HRP-conjugated anti-mouse or rabbit IgG secondary antibodies, an ECL system (Amersham Imager 600, Cytive, Marlborough, MA, USA) was used for detection.

### 4.9. GST-Pull-Down Assays Using GST-Af1521 Macrodomain and R5BD

GST-fused-Af1521 macrodomain and Rab5-binding domain (R5BD) were expressed in *Escherichia coli* BL21 Rosetta supercompetent cells (Merck Millipore, Burlington, MA, USA) for 16 h after addition of 1 mM IPTG at room temperature [46]. GST-Af1521 macrodomain and GST-R5BD were extracted from competent cells in PBS by sonication after addition of 1% Triton X-100 and were purified using Glutathione-Sepharose 4B according to the manufacturer’s instructions (Cytiva).

HeLa cells (3 × 10^5^ cells) seeded onto 6-well plates were incubated (1 day, 37 °C) in DMEM with 10% FBS. Cell lysates were prepared with lysis buffer containing 50 mM Tris-HCl (pH 7.4), 200 mM NaCl, 1 mM EDTA, 1% Triton X-100, 10% glycerol, 1 mM DTT, 10 μM PJ34, 1 μM ADP-HPD, and protease inhibitor cocktail. The resultant lysate was mixed with GST-Af1521 macrodomain and R5BD immobilized on Glutathione Sepharose 4B beads (4 h, 4 °C) on a rotating wheel. After washing three times with the lysis buffer, complexes were collected in LDS sample buffer.

### 4.10. Extraction of the Membrane Fraction and Lipid Isolation and Measurement of PI and PIP with Mass Spectrometry

Cells were precipitated with 0.5 M trichloroacetic acid (TCA). After washing with 5% TCA with 1 mM EDTA, membrane fractions were lysed with MeOH:CHCl_3_:12N HCl (80:40:1) and then separated by addition of CHCl3:0.1 N HCl (1:1). The organic layer was collected and concentrated using a vacuum evaporator. 9-Aminoacridine (Sigma-Aldrich, St. Louis, MI, USA) was used as a matrix. Mass spectrometry was performed using matrix-assisted laser desorption and ionization/time of flight mass spectrometry (MALDI-TOF-MS) (ultrafleXtreme, Bruker, Billerica, MA, USA) [47]. The mass spectra of phosphatidylinositol (PI) and phosphatidylinositol phosphate (PIP) were acquired in the negative ion reflector mode.

### 4.11. Statistical Analysis

Statistical analysis was performed using Sigmaplot 13 (Systat Software Inc., San Jose, CA, USA). Significance was determined using Student’s *t*-test between two samples or one-way and two-way ANOVA with post hoc Tukey’s test for three or more groups. Data are means ± S.E.M of values from the indicated number of experiments. *p* values < 0.05 were considered significant. All representative experiments were repeated three times.

## Data Availability

Not applicable.

## Supplementary Materials

The following supporting information can be downloaded at: https://www.mdpi.com/article/10.3390/ijms23147827/s1.

## References

[1] Fatokun A.A., Dawson V.L., Dawson T.M. (2014). Parthanatos: Mitochondrial-linked mechanisms and therapeutic opportunities. Br. J. Pharmacol..

[2] Andrabi S.A., Kim N.S., Yu S.W., Wang H., Koh D.W., Sasaki M., Klaus J.A., Otsuka T., Zhang Z., Koehler R.C. (2006). Poly(ADP-ribose) (PAR) polymer is a death signal. Proc. Natl. Acad. Sci. USA.

[3] Yu S.W., Wang H., Poitras M.F., Coombs C., Bowers W.J., Federoff H.J., Poirier G.G., Dawson T.M., Dawson V.L. (2002). Mediation of poly(ADP-ribose) polymerase-1-dependent cell death by apoptosis-inducing factor. Science.

[4] Ame J.C., Spenlehauer C., de Murcia G. (2004). The PARP superfamily. Bioessays.

[5] D’Amours D., Desnoyers S., D’Silva I., Poirier G.G. (1999). Poly(ADP-ribosyl)ation reactions in the regulation of nuclear functions. Biochem. J..

[6] Mashimo M., Moss J. (2016). Functional Role of ADP-Ribosyl-Acceptor Hydrolase 3 in poly(ADP-Ribose) Polymerase-1 Response to Oxidative Stress. Curr. Protein Pept. Sci..

[7] Verheugd P., Butepage M., Eckei L., Luscher B. (2016). Players in ADP-ribosylation: Readers and Erasers. Curr. Protein Pept. Sci..

[8] Mashimo M., Bu X., Aoyama K., Kato J., Ishiwata-Endo H., Stevens L.A., Kasamatsu A., Wolfe L.A., Toro C., Adams D. (2019). PARP1 inhibition alleviates injury in ARH3-deficient mice and human cells. JCI Insight.

[9] Mashimo M., Kato J., Moss J. (2013). ADP-ribosyl-acceptor hydrolase 3 regulates poly (ADP-ribose) degradation and cell death during oxidative stress. Proc. Natl. Acad. Sci. USA.

[10] Yu S.W., Andrabi S.A., Wang H., Kim N.S., Poirier G.G., Dawson T.M., Dawson V.L. (2006). Apoptosis-inducing factor mediates poly(ADP-ribose) (PAR) polymer-induced cell death. Proc. Natl. Acad. Sci. USA.

[11] Wang Y., Kim N.S., Haince J.F., Kang H.C., David K.K., Andrabi S.A., Poirier G.G., Dawson V.L., Dawson T.M. (2011). Poly(ADP-ribose) (PAR) binding to apoptosis-inducing factor is critical for PAR polymerase-1-dependent cell death (parthanatos). Sci. Signal.

[12] Wang Y., An R., Umanah G.K., Park H., Nambiar K., Eacker S.M., Kim B., Bao L., Harraz M.M., Chang C. (2016). A nuclease that mediates cell death induced by DNA damage and poly(ADP-ribose) polymerase-1. Science.

[13] Andrabi S.A., Umanah G.K., Chang C., Stevens D.A., Karuppagounder S.S., Gagne J.P., Poirier G.G., Dawson V.L., Dawson T.M. (2014). Poly(ADP-ribose) polymerase-dependent energy depletion occurs through inhibition of glycolysis. Proc. Natl. Acad. Sci. USA.

[14] Fouquerel E., Goellner E.M., Yu Z., Gagne J.P., Barbi de Moura M., Feinstein T., Wheeler D., Redpath P., Li J., Romero G. (2014). ARTD1/PARP1 negatively regulates glycolysis by inhibiting hexokinase 1 independent of NAD+ depletion. Cell Rep..

[15] Kam T.I., Mao X., Park H., Chou S.C., Karuppagounder S.S., Umanah G.E., Yun S.P., Brahmachari S., Panicker N., Chen R. (2018). Poly(ADP-ribose) drives pathologic alpha-synuclein neurodegeneration in Parkinson’s disease. Science.

[16] Koh D.W., Dawson T.M., Dawson V.L. (2005). Mediation of cell death by poly(ADP-ribose) polymerase-1. Pharmacol. Res. Off. J. Ital. Pharmacol. Soc..

[17] Lee Y., Karuppagounder S.S., Shin J.H., Lee Y.I., Ko H.S., Swing D., Jiang H., Kang S.U., Lee B.D., Kang H.C. (2013). Parthanatos mediates AIMP2-activated age-dependent dopaminergic neuronal loss. Nat. Neurosci..

[18] Eliasson M.J., Sampei K., Mandir A.S., Hurn P.D., Traystman R.J., Bao J., Pieper A., Wang Z.Q., Dawson T.M., Snyder S.H. (1997). Poly(ADP-ribose) polymerase gene disruption renders mice resistant to cerebral ischemia. Nat. Med..

[19] Andrabi S.A., Kang H.C., Haince J.F., Lee Y.I., Zhang J., Chi Z., West A.B., Koehler R.C., Poirier G.G., Dawson T.M. (2011). Iduna protects the brain from glutamate excitotoxicity and stroke by interfering with poly(ADP-ribose) polymer-induced cell death. Nat. Med..

[20] Khan I., Steeg P.S. (2021). Endocytosis: A pivotal pathway for regulating metastasis. Br. J. Cancer.

[21] Conner S.D., Schmid S.L. (2003). Regulated portals of entry into the cell. Nature.

[22] Grosshans B.L., Ortiz D., Novick P. (2006). Rabs and their effectors: Achieving specificity in membrane traffic. Proc. Natl. Acad. Sci. USA.

[23] Seabra M.C., Goldstein J.L., Sudhof T.C., Brown M.S. (1992). Rab geranylgeranyl transferase. A multisubunit enzyme that prenylates GTP-binding proteins terminating in Cys-X-Cys or Cys-Cys. J. Biol. Chem..

[24] Garrett M.D., Zahner J.E., Cheney C.M., Novick P.J. (1994). GDI1 encodes a GDP dissociation inhibitor that plays an essential role in the yeast secretory pathway. EMBO J..

[25] Gagne J.P., Isabelle M., Lo K.S., Bourassa S., Hendzel M.J., Dawson V.L., Dawson T.M., Poirier G.G. (2008). Proteome-wide identification of poly(ADP-ribose) binding proteins and poly(ADP-ribose)-associated protein complexes. Nucleic Acids Res..

[26] Teloni F., Altmeyer M. (2016). Readers of poly(ADP-ribose): Designed to be fit for purpose. Nucleic Acids Res..

[27] Lin X.P., Mintern J.D., Gleeson P.A. (2020). Macropinocytosis in Different Cell Types: Similarities and Differences. Membranes.

[28] Timinszky G., Till S., Hassa P.O., Hothorn M., Kustatscher G., Nijmeijer B., Colombelli J., Altmeyer M., Stelzer E.H., Scheffzek K. (2009). A macrodomain-containing histone rearranges chromatin upon sensing PARP1 activation. Nat. Struct. Mol. Biol..

[29] Qi Y., Liang Z., Wang Z., Lu G., Li G. (2015). Determination of Rab5 activity in the cell by effector pull-down assay. Methods Mol. Biol..

[30] Siddhanta U., McIlroy J., Shah A., Zhang Y., Backer J.M. (1998). Distinct roles for the p110alpha and hVPS34 phosphatidylinositol 3’-kinases in vesicular trafficking, regulation of the actin cytoskeleton, and mitogenesis. J. Cell Biol..

[31] Mishra A., Eathiraj S., Corvera S., Lambright D.G. (2010). Structural basis for Rab GTPase recognition and endosome tethering by the C2H2 zinc finger of Early Endosomal Autoantigen 1 (EEA1). Proc. Natl. Acad. Sci. USA.

[32] Cavalli V., Vilbois F., Corti M., Marcote M.J., Tamura K., Karin M., Arkinstall S., Gruenberg J. (2001). The stress-induced MAP kinase p38 regulates endocytic trafficking via the GDI:Rab5 complex. Mol. Cell.

[33] Zeigerer A., Gilleron J., Bogorad R.L., Marsico G., Nonaka H., Seifert S., Epstein-Barash H., Kuchimanchi S., Peng C.G., Ruda V.M. (2012). Rab5 is necessary for the biogenesis of the endolysosomal system in vivo. Nature.

[34] Zhu G., Zhai P., Liu J., Terzyan S., Li G., Zhang X.C. (2004). Structural basis of Rab5-Rabaptin5 interaction in endocytosis. Nat. Struct. Mol. Biol..

[35] Zhang Z., Zhang T., Wang S., Gong Z., Tang C., Chen J., Ding J. (2014). Molecular mechanism for Rabex-5 GEF activation by Rabaptin-5. eLife.

[36] Zhu G., Liu J., Terzyan S., Zhai P., Li G., Zhang X.C. (2003). High resolution crystal structures of human Rab5a and five mutants with substitutions in the catalytically important phosphate-binding loop. J. Biol. Chem..

[37] Merithew E., Hatherly S., Dumas J.J., Lawe D.C., Heller-Harrison R., Lambright D.G. (2001). Structural plasticity of an invariant hydrophobic triad in the switch regions of Rab GTPases is a determinant of effector recognition. J. Biol. Chem..

[38] Eathiraj S., Pan X., Ritacco C., Lambright D.G. (2005). Structural basis of family-wide Rab GTPase recognition by rabenosyn-5. Nature.

[39] Sigismund S., Confalonieri S., Ciliberto A., Polo S., Scita G., Di Fiore P.P. (2012). Endocytosis and signaling: Cell logistics shape the eukaryotic cell plan. Physiol. Rev..

[40] Sorkin A., von Zastrow M. (2009). Endocytosis and signalling: Intertwining molecular networks. Nat. Rev. Mol. Cell Biol..

[41] Taylor M.J., Perrais D., Merrifield C.J. (2011). A high precision survey of the molecular dynamics of mammalian clathrin-mediated endocytosis. PLoS Biol..

[42] Sun Q., Fan W., Chen K., Ding X., Chen S., Zhong Q. (2008). Identification of Barkor as a mammalian autophagy-specific factor for Beclin 1 and class III phosphatidylinositol 3-kinase. Proc. Natl. Acad. Sci. USA.

[43] Bohdanowicz M., Balkin D.M., De Camilli P., Grinstein S. (2012). Recruitment of OCRL and Inpp5B to phagosomes by Rab5 and APPL1 depletes phosphoinositides and attenuates Akt signaling. Mol. Biol. Cell.

[44] Navaroli D.M., Bellve K.D., Standley C., Lifshitz L.M., Cardia J., Lambright D., Leonard D., Fogarty K.E., Corvera S. (2012). Rabenosyn-5 defines the fate of the transferrin receptor following clathrin-mediated endocytosis. Proc. Natl. Acad. Sci. USA.

[45] Boothe T., Lim G.E., Cen H., Skovso S., Piske M., Li S.N., Nabi I.R., Gilon P., Johnson J.D. (2016). Inter-domain tagging implicates caveolin-1 in insulin receptor trafficking and Erk signaling bias in pancreatic beta-cells. Mol. Metab..

[46] Mashimo M., Onishi M., Uno A., Tanimichi A., Nobeyama A., Mori M., Yamada S., Negi S., Bu X., Kato J. (2021). The 89-kDa PARP1 cleavage fragment serves as a cytoplasmic PAR carrier to induce AIF-mediated apoptosis. J. Biol. Chem..

[47] Wenk M.R., Lucast L., Di Paolo G., Romanelli A.J., Suchy S.F., Nussbaum R.L., Cline G.W., Shulman G.I., McMurray W., De Camilli P. (2003). Phosphoinositide profiling in complex lipid mixtures using electrospray ionization mass spectrometry. Nat. Biotechnol..

[48] Aoyama M., Sun-Wada G.H., Yamamoto A., Yamamoto M., Hamada H., Wada Y. (2012). Spatial restriction of bone morphogenetic protein signaling in mouse gastrula through the mVam2-dependent endocytic pathway. Dev. Cell.

[49] Takasuga S., Horie Y., Sasaki J., Sun-Wada G.H., Kawamura N., Iizuka R., Mizuno K., Eguchi S., Kofuji S., Kimura H. (2013). Critical roles of type III phosphatidylinositol phosphate kinase in murine embryonic visceral endoderm and adult intestine. Proc. Natl. Acad. Sci. USA.

