# Supplementary material for: Poly(ADP-ribose) Polymerase 1 Mediates Rab5 Inactivation after DNA Damage
Source: Int J Mol Sci. 2022 Jul 15;23(14):7827. doi: 10.3390/ijms23147827 (PMC9319841; doi:10.3390/ijms23147827)
Supplement: Supplementary file 1 [file ijms-23-07827-s001.zip › ijms-1789104-supplementary.pdf]

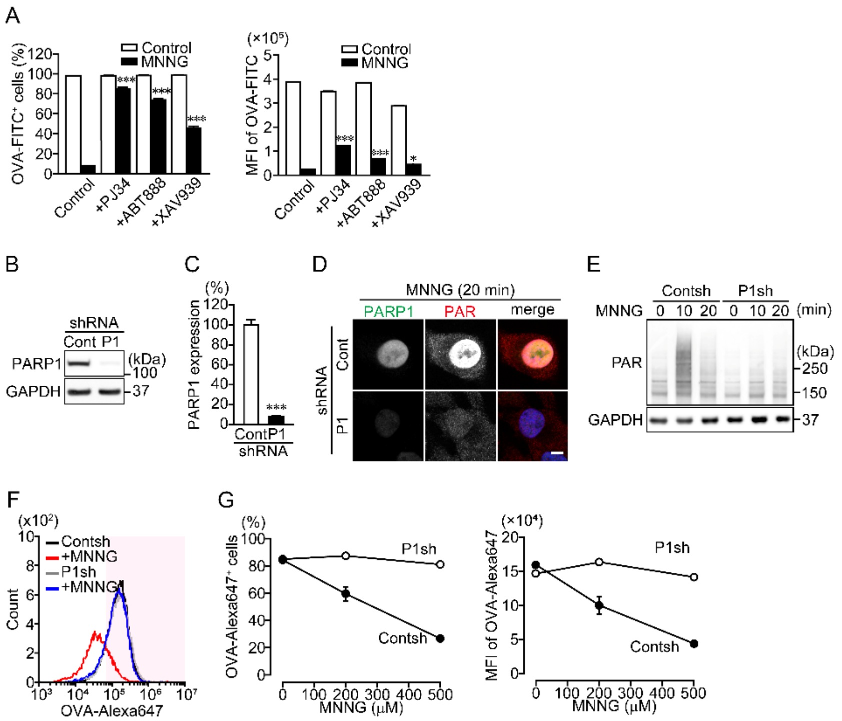

Figure S1. PARP1 activation inhibits OVA uptake in NIH3T3 cells and HeLa cells. (A) Effect of PARP inhibitors on OVA-FITC uptake (the percentages of OVA-FITC+ cells (left) and MFI of OVA-FITC in NIH3T3 cells (right)) following exposure to MNNG in NI3T3 cells. NIH3T3 cells were exposed to MNNG (500 μM, 20 min) before incubation with OVA-FITC (50 μg/ml, 1 h) and chased with OVA (100 μg/ml, 1 h). Cells were pretreated with or without PARP inhibitors (10 μM) for 10 min. Shown are means ± SEM (n = 3). $^{*}P < 0.05$, $^{***}P < 0.001$ vs. control group exposed to MNNG. (B) PARP1 expression in HeLa cells stably expressing PARP1 shRNA. (C) Relative PARP1 expression. PARP1 protein levels were normalized to GAPDH. Shown are means ± SEM (n = 3). $^{***}P < 0.001$. (D) Subcellular localization of PARP1 and MNNG-induced PAR synthesis. After exposure to MNNG (100 μM, 20 min), HeLa cells were stained with anti-PARP1 (green) and anti-PAR (red) antibodies. Nuclei were stained with DAPI (blue). Scale bar: 10 μm. (E) Effect of PARP1 shRNA on PAR synthesis. After exposure to MNNG (100 μM) for the indicated times, HeLa cells were subjected to Western blotting. (F) OVA-Alexa647 uptake. HeLa cells were exposed to MNNG (100 μM, 20 min) before incubation with OVA-Alexa647 (50 μg/ml, 1 h) and chased with OVA (100 μg/ml, 1 h). OVA-Alexa647+ cells (magenta region) were determined from the results in the absence of OVA-Alexa647. (G) MNNG concentration-dependent inhibition of OVA-Alexa647 uptake (the percentages of OVA-Alexa647+ cells (left) and MFI of OVA-Alexa647 in HeLa cells (right)). Shown are means ± SEM (n = 3). $P < 0.001$ at > 200 μM. Data information: Panels (A), (E): two-way ANOVA with *post hoc* Tukey's test; (C): Student's *t*-test.

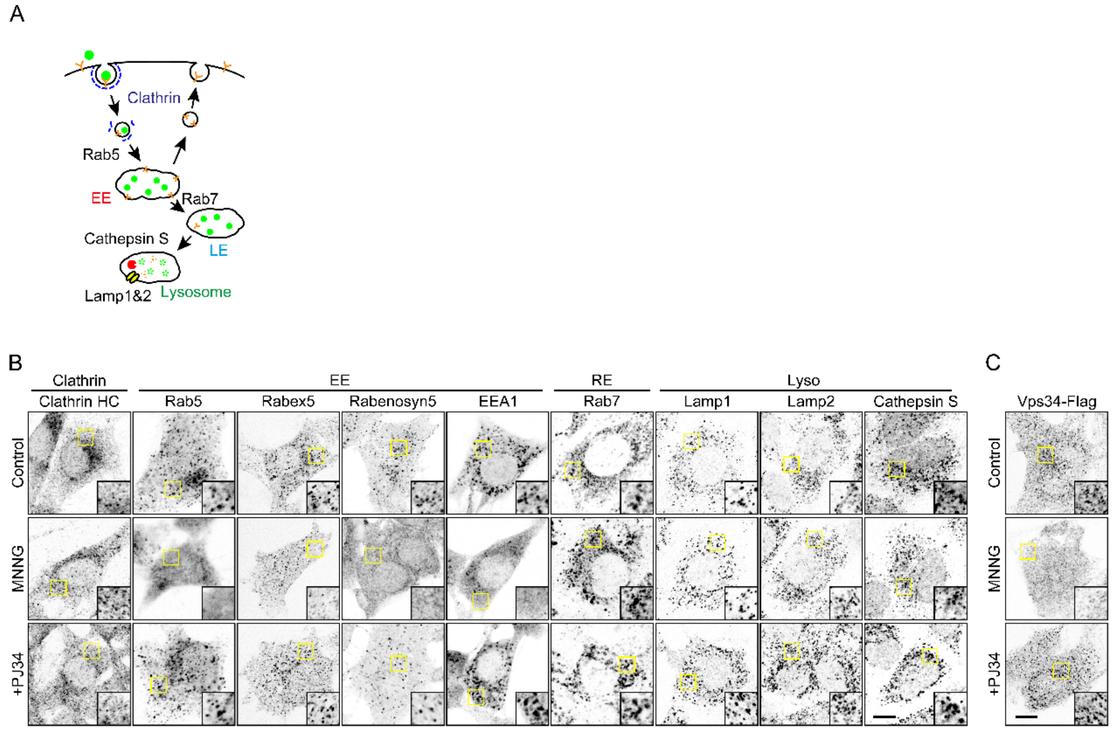

Figure S2. PARP1 activation facilitates the release of Rab5 and EEA1 from EE after DNA damage. (A) Endocytosis pathway. (B) Subcellular localization of proteins involved in endocytosis. After exposure to MNNG (500 μM, 20 min), NIH3T3 cells were pretreated for 10 min with or without PJ34 (10 μM) then stained with indicated antibodies. Scale bar: 10 μm. (C) Subcellular localization of Vps34-Flag following exposure to MNNG. After exposure to MNNG (500 μM, 20 min), NIH3T3 cells were stained with anti-Flag antibodies. Scale bar; 10 μm.

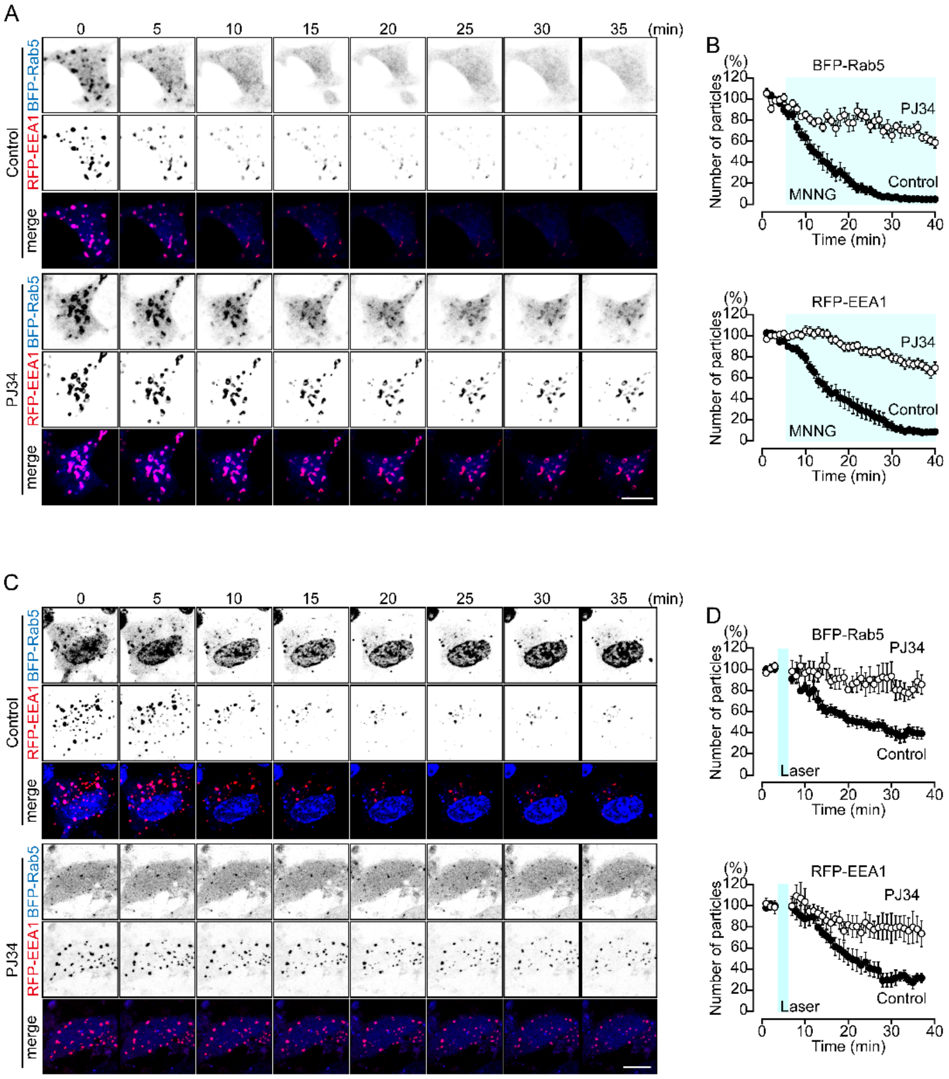

Figure S3. Time-lapse imaging of BFP-Rab5 and RFP-EEA1 following exposure to MNNG. (A) Subcellular localization of BFP-Rab5 and RFP-EEA1 following exposure to MNNG. NIH3T3 cells expressing BFP-Rab5 and RFP-EEA1 were pretreated for 10 min with or without PJ34 (10 µM) then exposed to MNNG (500 µM). Scale bar: 10 µm. (B) Numbers of BFP-Rab5 and RFP-EEA1 particles. NIH3T3 cells were exposed to MMNG beginning 3 min after the start of imaging (blue region). Shown are means ± SEM (n = 7-13). (C) Subcellular localization of BFP-Rab5 and RFP-EEA1 following micro-irradiation-induced DNA damage. NIH3T3 cells expressing BFP-Rab5 (blue) and RFP-EEA1 (red) were pretreated for 10 min with or without PJ34 (10 µM), after which the nuclei were irradiated using a 405 nm laser in the presence of Hoechst 33258. Scale bar: 10 µm. (D) Numbers of BFP-Rab5 and RFP-EEA1 particles. Laser irradiation was initiated 3 min after the start of imaging (blue region). Shown are means ± SEM (n = 5-8).

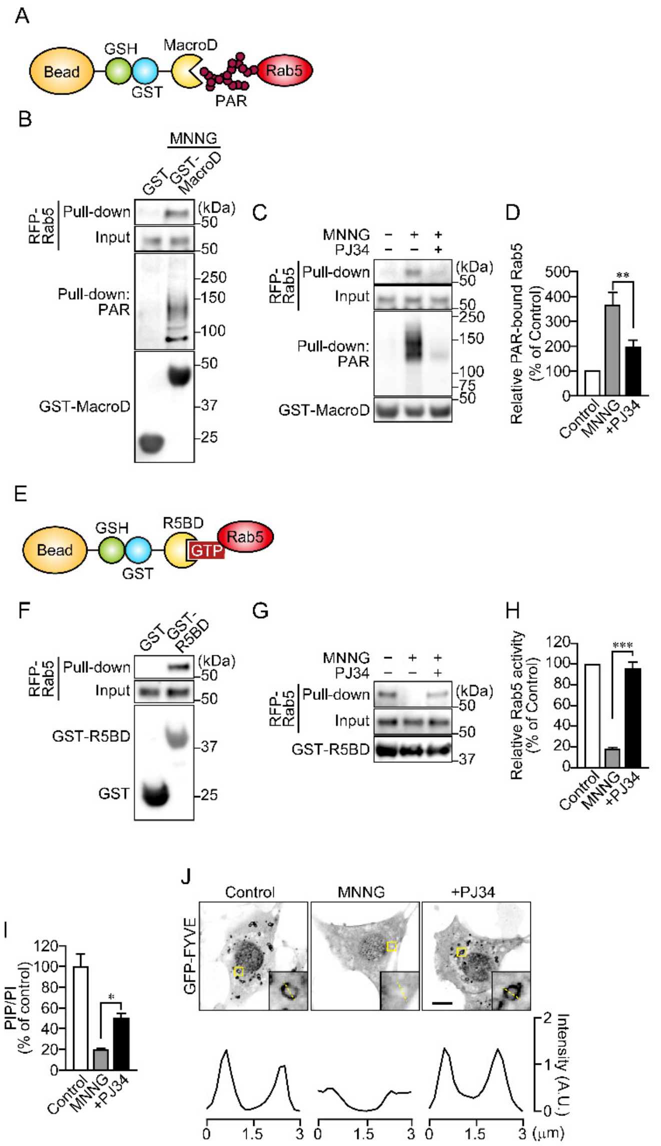

Figure S4. PAR-binding to Rab5 suppresses Rab5 activity, decreasing PI3P levels in endosomes. (A) GST-Af1521 macrodomain. (B) PAR-binding assays. After exposure to MNNG (100 μM, 10 min), HeLa cells expressing RFP-Rab5 were subjected first to pull-down assays using GST or GST-Af1521 macrodomain and then to Western blotting using anti-mCherry (RFP), anti-GST, and anti-PAR antibodies. (C) Effect of PJ34 on PAR-binding to Rab5 following exposure to MNNG. After exposure to MNNG (100 μM, 10 min), HeLa cells expressing RFP-Rab5 were subjected first to a pull-down assay using GST-macrodomain and then to Western blotting using anti-mCherry, anti-GST, and anti-PAR antibodies. (D) Relative levels of PAR-bound Rab5. Ratio of PAR-bound Rab5 (pull-down) to Rab5 (input) were normalized to control. Shown are means ± SEM (n = 3). $^{**}P < 0.01$. (E) GST-R5BD. (F) Rab5 activity assays. After exposure to MNNG (100 μM, 20 min), HeLa cells expressing RFP-Rab5 were subjected first to pull-down assays using GST or GST-R5BD and then to Western blotting using anti-mCherry and anti-GST antibodies. (G) Effect of PARP inhibition on Rab5 activity following exposure to MNNG. HeLa cells expressing RFP-Rab5 were pretreated for 10 min with or without PJ34 (10 μM) before exposure to MNNG (100 μM, 20 min), after which they were subjected first to pull-down assays using GST-R5BD and then the Western blotting using anti-mCherry and anti-GST antibodies. (H) Relative Rab5 activity. Ratios of GTP-bound Rab5 (pull-down) to Rab5 (input) were normalized to control. Shown are means ± SEM (n = 3). $^{***}P < 0.001$ (I) Relative PIP/PI levels. Ratios of PI to PIP were normalized to control. Cells were pretreated for 10 min with or without PJ34 (10 μM). Shown are means ± SEM (n = 3). $^{*}P < 0.05$ (J) Subcellular localization of GFP-FYVE. NIH3T3 cells were pretreated for 10 min with or without PJ34 (10 μM) then exposed to MNNG (500 μM, 20 min). Graphs below show the

fluorescence intensity of GFP-FYVE on the dashed yellow lines in the magnified images. Scale bar: 10 μm. Data information: Panels (D), (H), (I): one-way ANOVA with *post hoc* Tukey's test.

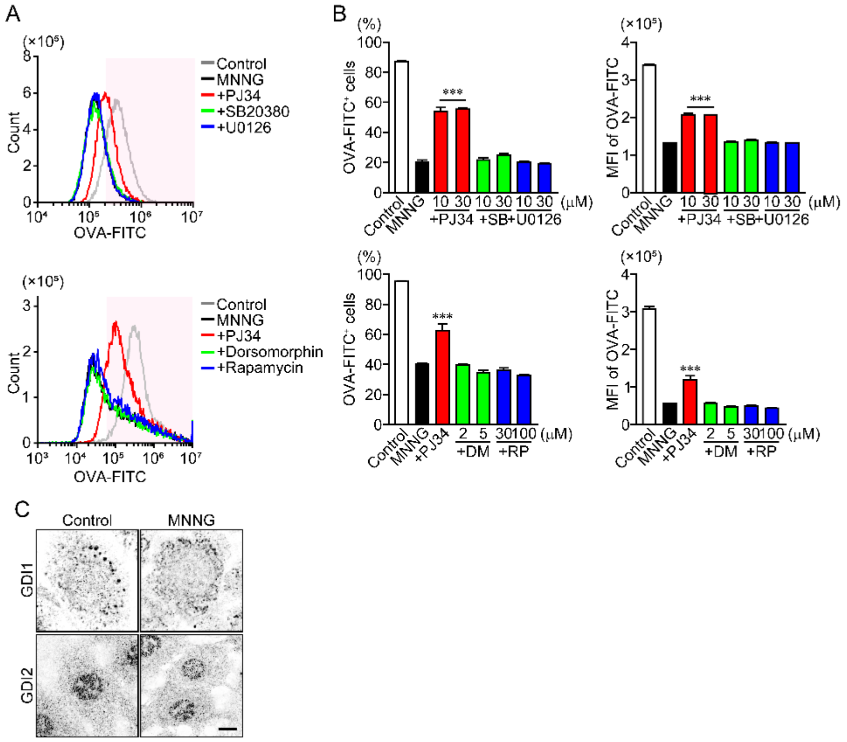

Figure S5. GDI does not participate in PARP1-mediated inhibition of OVA uptake. (A) Effect of kinase inhibitors on OVA-FITC uptake following exposure to MNNG. NIH3T3 cells were pretreated for 10 min with or without PJ34, SB203580 (SB), U0126, dorsomorphin (DM), or rapamycin (RP) at indicated concentrations and then exposed to MNNG (500 μM, 20 min), after which they were incubated with OVA-FITC (50 μg/ml, 1 h) and chased with OVA (100 μg/ml, 1 h). OVA-FITC$^+$ cells (magenta region) were counted using flow cytometry. (B) OVA-FITC uptake (the percentages of OVA-FITC$^+$ cells (left) and MFI of OVA-FITC in NIH3T3 cells (right)). Shown are means ± SEM (n = 3). $^{***}P < 0.001$ vs. MNNG. (C) Effect of kinase inhibitors on the subcellular localization of GDI1 and GDI2 following exposure to MNNG. After exposure to MNNG (500 μM, 20 min), NIH3T3 cells were stained with anti-GDI1 and GDI2 antibodies. Scale bar: 10 μm. Data information: Panel (B): two-way ANOVA with *post hoc* Tukey's test.
